# Association between Subjective Indicators of Recovery Status and Heart Rate Variability among Divison-1 Sprint-Swimmers

**DOI:** 10.3390/sports6030093

**Published:** 2018-09-11

**Authors:** Andrew A. Flatt, Michael R. Esco, Fabio Y. Nakamura

**Affiliations:** 1 Department of Health Sciences and Kinesiology, Biodynamics and Human Performance Center, Georgia Southern University, 11935 Abercorn St., Savannah, GA 31419, USA; 2 Department of Kinesiology, University of Alabama, Tuscaloosa, AL 35487, USA; mresco@ua.edu; 3 Department of Medicine and Aging Sciences, University of Chieti-Pescara, 66100 Chieti, Italy; fabioy_nakamura@yahoo.com.br; 4 The College of Healthcare Sciences, James Cook University, Townsville, QLD 4811, Australia

**Keywords:** autonomic, parasympathetic, cardiovascular, sports science, wellness

## Abstract

Heart rate variability (HRV) is a physiological marker of training adaptation among athletes. However, HRV interpretation is challenging when assessed in isolation due to its sensitivity to various training and non-training-related factors. The purpose of this study was to determine the association between athlete-self report measures of recovery (ASRM) and HRV throughout a preparatory training period. Ultra-short natural logarithm of the root mean square of successive differences (LnRMSSD) and subjective ratings of sleep quality, fatigue, muscle soreness, stress and mood were acquired daily for 4 weeks among Division-1 sprint-swimmers (*n* = 17 males). ASRM were converted to *z*-scores and classified as average (*z*-score −0.5–0.5), better than average (*z*-score > 0.5) or worse than average (*z*-score < −0.5). Linear mixed models were used to evaluate differences in LnRMSSD based on ASRM classifications. LnRMSSD was higher (*p* < 0.05) when perceived sleep quality, fatigue, stress and mood were better than average versus worse than average. Within-subject correlations revealed that 15 of 17 subjects demonstrated at least one relationship (*p* < 0.05) between LnRMSSD and ASRM variables. Changes in HRV may be the result of non-training related factors and thus practitioners are encouraged to include subjective measures to facilitate targeted interventions to support training adaptations.

## 1. Introduction

Resting heart rate variability (HRV) is considered a global marker of homeostasis [1] and is widely implemented as an indicator of training adaptation in the applied sports setting [2,3,4,5,6]. For example, HRV has been shown to reflect changes in performance [4] and training load [7] among competitive swimmers. However, interpretation of HRV responses to training and competition have been demonstrated to be context dependent [8]. Factors such as training type and intensity [9], training phase [10], proximity to competition [10], fitness level [9] and body mass [11] have all been shown to influence HRV responses. Thus, establishing whether a change in HRV can be interpreted as positive or negative should be considered alongside contextual factors as described above and in addition to other markers of fatigue and recovery status [12].

The use of athlete self-report measures (ASRM) in the form of brief wellness questionnaires provide a convenient and effective means of monitoring an athlete’s perceptual response to training [13]. Decrements in ASRM are strong indicators of a maladaptive training response that have been associated with overtraining [14]. Hooper et al. demonstrated that a combination of both physiological (i.e., autonomic) and ASRM accurately predicted changes in swim performance in response to tapering [15]. In addition, Tian et al. reported that psycho-social stressors (not quantified) contributed to the development of non-functional overreaching and altered HRV in elite wrestlers, demonstrating how non-training related factors may have meaningful effects on training responses [16]. Thus, evaluation of an athlete’s response to training may be enhanced by considering both physiological (e.g., HRV) and ASRM together [8,12].

It is well established that HRV is sensitive to a variety of physiological (e.g., hemodynamic, endocrine, thermoregulatory), environmental and perceived psychological factors [1]. Arterial baroreflexes, activity of the hypothalamic-pituitary-adrenocortical axis and renin-angiotensin-aldosterone system as well as concentrations of thyroid and sex hormones have all been shown to affect HRV [1]. Apart from physical training, variables such as sleep quality, stress and mood can also affect physiological parameters and have been associated with different HRV patterns in cross-sectional studies [17,18,19]. For example, Werner et al., found that higher objective and subjective sleep quality were associated with higher post-waking, vagally-mediated HRV among a sample of healthy college students [17]. Additionally, vagal-indexes of HRV have been shown to demonstrate an inverse relationship with markers of mental stress [18]. Thus, changes in HRV that are interpreted to be undesirable (e.g., indicative of fatigue) may be caused by non-training related factors, which can be communicated via ASRM. In this context, the appropriate intervention for a fatigued athlete may be to address life-stressors or sleep quality issues rather than only adjusting training load to improve autonomic activity and support positive adaptation to training.

Limited research exists that evaluates associations between HRV and ASRM among swimmers over a longitudinal training period. It has been demonstrated that significant increases in high-intensity swimming load are associated with decrements in both HRV and ASRM [7]. However, establishing associations between HRV and perceptual training responses, uninfluenced by significant changes in training load are required to further understand how ASRM relate with HRV under normal conditions in swimmers. This information may assist practitioners in interpreting HRV responses which may facilitate interventions that target underlying issues contributing to the undesirable training response, which may be training or life-style related. Therefore, the purpose of this study was to determine the association between HRV and ASRM among collegiate sprint-swimmers throughout standardized, preparatory training. We hypothesized that lower ASRM would be associated with decrements in vagally-mediated HRV.

## 2. Materials and Methods

### 2.1. Participants

Sprint-swimmers (*n* = 17 males, age = 21.6 ± 1.8 years, height = 187.5 ± 9.2 cm, weight = 84.6 ± 6.0 kg, competitive experience = 11.2 ± 4.4 years) from a Division 1 National Collegiate Athletic Association (NCAA) program volunteered for this study, five of whom were Olympians. Ethical approval was granted from the Institutional Review Board. All swimmers provided written informed consent prior to participation in this study.

### 2.2. Procedures

#### 2.2.1. Observation Period

The swimmers were monitored over a 4-week preparatory period at the beginning of the fall academic semester. As all participating swimmers were members of the same collegiate team under the same head coach, training was largely standardized. Weekly training consisted of 19.5 h of total training time including three, 60-min resistance training sessions and nine, 90–120-min pool sessions. The total planned volume load in swimming distance covered over the 4-week period was 136.6 km with a weekly total distance coefficient of variation of 12.4%. While training was progressive in nature, there was no systematic overload or taper during the observation period.

#### 2.2.2. Heart Rate Parameters

All subjects were provided with a smartphone application and pulse-wave finger sensor (ithlete^TM^, HRVfit LTD., Southampton, UK) for daily HRV measures. These materials have been shown to provide acceptable agreement with simultaneous electrocardiograph recordings in healthy and athletic populations under supine, seated and standing positions [20]. Over four consecutive weeks, HRV was measured daily in the seated position by the subjects after waking and elimination (between 5:30 and 8:30 a.m.), following the same procedures used in a previous investigation [7]. Briefly, pulse-rate was recorded for 1-min via the application preceded by a 1-min stabilization period while the subjects were seated comfortably and motionless and breathed naturally [21]. The HR parameters evaluated in the current study include resting heart rate (RHR) and the natural logarithm of the root mean square of successive differences (LnRMSSD). LnRMSSD is an accepted marker of cardiac-parasympathetic activity and is the preferred HRV metric for field-based monitoring [8]. The LnRMSSD value is multiplied by twenty by the application to fit an approximate 100-point scale for simplified interpretation. A built-in processing algorithm described previously controls for artifacts and ectopic beats [7]. Immediately following an HRV recording, data were automatically uploaded to a web-based software for analysis by the researchers.

#### 2.2.3. Athlete Self-Report Measures

Following the HRV recording, subjects then completed a brief wellness questionnaire adapted from McLean et al. on the application where they provided subjective ratings of their sleep quality (1 = Insomnia, 5 = Okay, 9 = Very Restful), fatigue (1 = Always Tired, 5 = Okay, 9 = Very Fresh), muscle soreness (1 = Very Sore, 5 = Okay, 9 = Feeling Great), stress (1 = Very Stressed, 5 = Okay, 9 = Very Relaxed) and mood (1 = Irritable, 5 = Okay, 9 = Very Positive) on a 9-point sliding scale [22]. These well-being categories are consistent with those used previously to monitor training responses in swimmers [14]. Upon completion of the wellness survey, data were automatically uploaded to the web-based software for analysis.

### 2.3. Statistical Analysis

Multicollinearity among ASRM *z*-scores was assessed with Spearman’s ρ. Multicollinearity was defined as a correlation coefficient >0.5 [23]. Subjective indicators of recovery status were converted to *z*-scores for each individual and categorized as “average” (*z*-score within −0.5–0.5), “better” (*z*-score > 0.5) or “worse” (*z*-score < −0.5). There were 28 observations (i.e., 4 weeks) for each swimmer. Linear mixed models were used to evaluate variation in HR-parameters according to whether subjective indicators of recovery were average, better than average or worse than average using the *z*-score thresholds described above. The subjective classification (average, better or worse) was included as a within-subjects repeated measure and swimmer identification was included as a random effect. Tukey’s Honest Significant Difference tests were used for post-hoc analyses. In addition, Cohen’s effect size ± 90% confidence interval (ES ± 90% CI) were calculated to evaluate the magnitude of differences in HR-parameters among subjective classifications [24]. ES were interpreted qualitatively as follows: <0.2 was trivial, <0.6 was small, <1.2 was moderate, <2.0 was large, and >2.0 was very large [25]. The effect was deemed unclear when the 90% CI crossed the threshold for both substantially positive (0.2) and negative (−0.2) values [26]. Within-subject correlations between HR-parameters and ASRM *z*-scores were quantified using Spearman’s ρ and interpreted as: <0.1, trivial; 0.1–0.29, small; 0.3–0.49, moderate; 0.5–0.7, large; 0.7–0.89, very large; >0.9 nearly perfect [25]. *p* values <0.05 were considered statistically significant. Statistical procedures were performed using JMP Pro 12 (SAS Institute Inc., Cary, NC, USA) and Excel 2016 (Microsoft Corp., Redmond, WA, USA).

## 3. Results

### 3.1. HR-Parameters

#### 3.1.1. RHR

A significant main effect was observed for sleep (F_2,14_ = 8.171, *p* = 0.001). RHR was lower with better sleep compared with worse sleep (*p* < 0.001). A significant main effect was observed for stress (F_2,14_ = 4.399, *p* = 0.022). RHR was lower with better stress compared with worse stress (*p* = 0.020). A significant main effect was observed for mood (F_2,14_ = 6.494, *p* = 0.005). RHR was lower with better mood compared with average (*p* = 0.015) and worse mood (*p* = 0.011). No main effects were observed for fatigue (F_2,14_ = 2.302, *p* = 0.122) or soreness (F_2,14_ = 0.382, *p* = 0.686).

#### 3.1.2. LnRMSSD

A significant main effect was observed for sleep (F_2,14_ = 14.409, *p* < 0.0001). LnRMSSD was higher with better sleep compared with average (*p* = 0.023) and worse sleep (*p* < 0.0001). Additionally, LnRMSSD with average sleep was higher than with worse sleep (*p* = 0.027). A significant main effect was observed for fatigue (F_2,14_ = 10.112, *p* < 0.001). LnRMSSD was higher with better fatigue compared with worse fatigue (*p* < 0.001). A significant main effect was observed for stress (F_2,14_ = 4.509, *p* = 0.019). LnRMSSD was higher with better stress compared with worse stress (*p* = 0.014). A significant main effect was observed for mood (F_2,14_ = 10.436, *p* < 0.001). LnRMSSD was higher with better mood compared with average (*p* = 0.027) and worse mood (*p* < 0.001). No main effect was observed for soreness (F_2,14_ = 0.311, *p* = 0.736).

**Figure 1:**
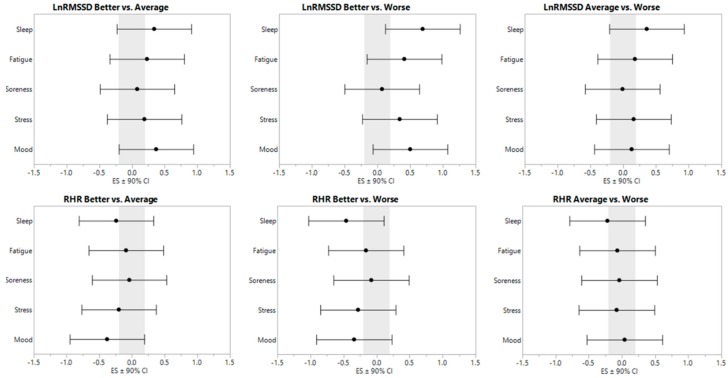
Effect sizes ± 90% confidence interval for resting heart rate parameters relative to subjective categorization.

LnRMSSD and RHR model-adjusted, least-square values are displayed in Table 1. ES ± 90% CI are displayed in Figure 1.

#### 3.1.3. Within-Subject Correlations

Individual correlation coefficients between HR-parameters and ASRM *z*-scores are presented in Table 2. At least one significant correlation was observed in 15 of 17 swimmers for LnRMSSD and 8 of 17 swimmers for RHR.

#### 3.1.4. Multicollinearity 

Multicollinearity coefficients are presented in Table 3. Multicollinearity was observed between perceived ratings of stress and mood (ρ = 0.52). All other coefficients were ρ < 0.5. 

## 4. Discussion

The purpose of this study was to determine the association between HRV and ASRM among collegiate sprint-swimmers throughout standardized training, absent of substantial alterations in training load. The main finding was that LnRMSSD was significantly higher when perceived sleep quality, fatigue, stress and mood were better than average (*z*-score > 0.5) versus worse than average. In addition, RHR was significantly lower when perceived sleep quality, stress and mood were rated as better than average versus worse than average. 

Perceived sleep quality demonstrated the strongest association with cardiac-autonomic parameters. While subjective sleep quality is commonly used as an alternative to objective measures in athletes [27], it is unclear whether sleep disturbances, insufficient sleep or some other sleep quality indicator were influencing the subjective ratings in the current study. Nevertheless, both objective and subjective sleep quality have been associated with HRV in cross-sectional studies [17]. In addition, improvements in LnRMSSD and perceived sleep quality were observed in high-level swimmers throughout a training microcycle involving a cold water immersion recovery intervention [28]. Furthermore, Zhang et al. showed that shorter time in bed, less sleep time, longer sleep onset latency and lower sleep efficiency were all correlated with higher 24-h urinary epinephrine and norepinephrine [29]. Vgontzas et al. reported increased pro-inflammatory cytokines (interleukin-6 and tumor necrosis factor alpha) with modest (8 h vs. 6 h) sleep restriction [30]. These aforementioned physiological effects consequent of poor sleep may contribute to withdrawn cardiac-vagal activity and as a result, reduced LnRMSSD.

Decreased vagally-mediated HRV is commonly observed among individuals with chronic fatigue syndrome [31], a disorder characterized by unexplained extreme fatigue or tiredness. Our finding of an association between LnRMSSD and perceived fatigue is in support of Schmitt et al. who reported that a reduction in high-frequency spectral power derived from ~8-min recordings was the most commonly observed change in HRV associated with perceived fatigue among elite endurance athletes, monitored non-daily over a 4-year period [5]. Several other studies have demonstrated that significant increments in training load resulted in decrements (i.e., worsening) in both vagally-mediated HRV and perceived fatigue in a variety of athletes [4,7,32,33]. However, contrasting findings of increased LnRMSSD in association with greater perceived fatigue have been reported in endurance athletes throughout three weeks of overload training [6]. This disparity is explained by the fact that training-induced fatigue can manifest into Addisonic (sympathetic) or Basedowic (parasympathetic) symptoms [34] and reinforces the need to interpret cardiac-autonomic responses with ASRM for accurate interpretation [12].

The sympatho-adrenal medullary and hypothalamic-pituitary-adrenocortical axes mediate the physiological response to stress by modulating parasympathetic and sympathetic activity [35]. Not surprisingly, perceived stress and mood were inversely associated with HRV in the current study as well as in a variety of cross-sectional studies [18], likewise with mood disturbance [19]. Multicollinearity was observed between perceived stress and mood (Table 3), indicating that these two perceptions likely influence one another. In addition, the vagueness of the stress category prevents delineation of potential causes and sources of stress experienced by the swimmers. For example, this observation period overlapped with mid-term academic exams, a known stressor shown to reduce HRV [36]. In practice, higher perceived stress ratings should be followed up with direct communication with the athlete to identify the source of stress so that efforts can be made to mitigate its persistence.

The lack of association between perceived muscle soreness and HR-parameters is in agreement with a previous investigation in adult male soccer players who found no significant correlation between HRV and creatine kinase (a marker of muscle damage) during a training camp in the heat [37]. Moreover, Chen et al. found that vagally-mediate HRV was unrelated to pain and circulating muscle creatine kinase levels following an intense resistance training session in elite weightlifters [38]. Thus, practitioners should be aware that athletes may be experiencing muscle soreness despite LnRMSSD being at or above baseline, highlighting a limitation of HRV as a complete marker of recovery status.

Despite a strong inverse relationship between RHR and LnRMSSD, LnRMSSD demonstrated stronger associations with ASRM at both the group and individual level. Previous investigations have demonstrated that ultra-short LnRMSSD was more sensitive than RHR for reflecting responses [3] and fitness changes [39] to training among team-sport athletes. Despite a greater sensitivity to training and perceptual responses, interpreting LnRMSSD in conjunction with RHR is still encouraged for other reasons, such as detecting parasympathetic saturation [10].

A limitation of the current study was the selected *z*-score classifications for average, better than average and worse than average ASRM ratings. However, we are unaware of any published research that provides specific recommendations for this type of analysis. Other possible limitations include the sample size and observational study design. Finally, this study was also limited by the wellness questionnaire which does not provide specific information regarding sources of stress, cause of sleep or mood disturbance and so forth. However, we argue that keeping the questionnaire brief may enhance compliance with daily assessment and provides practitioners with an opportunity to engage the athlete in conversation regarding questionnaire results and their impact on HRV and recovery. 

## 5. Conclusions

In conclusion, our results indicate that during preparatory training, in the absence of systematic overload, higher vagally-mediated HRV was associated with more favorable perceptual training status indicators among high-level collegiate sprint-swimmers. These results add to the current body of knowledge by demonstrating a relationship between ASRM and LnRMSSD among competitive swimmers when assessed daily via smartphone-derived, ultra-short recordings and a brief wellness questionnaire. Finally, this study demonstrates that changes in HRV may be associated with perceived sleep quality, fatigue, stress or mood, which may have implications for making targeted interventions when decrements in HRV are observed in athletes. Practitioners are therefore encouraged to include subjective measures when interpreting physiological markers such as HRV for evaluating adaptation to training in swimmers.

## Figures and Tables

**Table 1:** Model-adjusted mean ± standard deviation for resting heart rate parameters.

Parameter	Better	Average	Worse
RHR (b·min^−1^)			
Sleep	62.0 ± 7.7 *	63.9 ± 7.7	65.6 ± 7.8
Fatigue	63.0 ± 7.7	63.6 ± 7.8	64.2 ± 7.8
Soreness	63.2 ± 7.8	63.5 ± 7.7	63.8 ± 7.7
Stress	62.3 ± 7.7 *	63.9 ± 7.8	64.5 ± 7.7
Mood	61.8 ± 7.7 *^¥^	64.7 ± 7.8	64.3 ± 7.6
LnRMSSD			
Sleep	86.6 ± 7.5 *^¥^	84.1 ± 7.5 *	81.3 ± 7.6
Fatigue	85.9 ± 7.9 *	84.1 ± 7.9	82.7 ± 7.9
Soreness	84.8 ± 7.8	84.2 ± 7.7	84.2 ± 7.8
Stress	85.7 ± 7.8 *	84.3 ± 7.9	83.0 ± 7.8
Mood	86.6 ± 7.7 *^¥^	83.7 ± 7.8	82.7 ± 7.7

RHR = resting heart rate; LnRMSSD = natural logarithm of the root mean square of successive differences. * = different than worse (*p* < 0.05). ^¥^ = different than average (*p* < 0.05).

**Table 2:** Individual correlation coefficients (Spearman’s ρ) between subjective indicators (*z*-scores) and resting heart rate (RHR) and natural logarithm of the root mean square of successive differences (LnRMSSD).

Parameter	Subject	Sleep	Fatigue	Soreness	Stress	Mood
**RHR (b·min^−1^)**	A	−0.56 **	−0.44 *	−0.51 **	−0.60 **	−0.46 *
	B	−0.22	−0.20	0.11	−0.33	−0.05
	C	−0.63 **	−0.09	0.21	0.19	−0.20
	D	−0.67 **	−0.06	−0.01	−0.05	−0.35
	E	−0.27	−0.09	0.45 *	−0.35	−0.31
	F	−0.20	−0.14	−0.06	−0.30	0.19
	G	0.18	−0.12	−0.30	0.17	0.03
	H	−0.58 **	0.11	−0.17	−0.38 *	−0.35
	I	−0.14	−0.27	−0.10	0.04	−0.26
	J	0.05	0.06	0.15	−0.02	0.06
	K	−0.02	−0.13	−0.12	−0.44 *	−0.27
	L	0.03	−0.05	0.09	−0.01	−0.22
	M	−0.24	0.20	0.21	−0.07	−0.10
	N	0.23	0.12	−0.14	0.29	0.14
	O	−0.36	−0.19	−0.14	−0.22	−0.35
	P	−0.41 *	−0.36	−0.30	−0.22	−0.41 *
	Q	−0.07	−0.49 **	−0.27	−0.16	−0.07
**LnRMSSD**	A	0.49 **	0.46 *	0.27	0.55 **	0.37 *
	B	0.06	0.49 **	0.01	0.43 *	0.25
	C	0.61 **	0.08	−0.26	−0.11	0.36
	D	0.69 **	0.24	0.02	0.12	0.25
	E	0.43 *	0.27	0.01	0.45 *	0.53 **
	F	0.04	0.25	−0.13	0.01	0.15
	G	0.21	0.14	0.23	0.39 *	0.28
	H	0.58 **	−0.08	0.03	0.45 *	0.53 **
	I	0.35	0.53 **	0.09	0.13	0.18
	J	−0.06	−0.16	−0.16	−0.23	−0.22
	K	0.10	0.23	0.15	0.39 *	0.25
	L	0.65 **	0.74 **	−0.05	0.10	0.41 *
	M	0.47 *	−0.15	−0.22	0.36	0.19
	N	0.07	0.24	0.40 *	−0.08	0.02
	O	0.32	0.34	0.15	0.23	0.50 **
	P	0.48 **	0.30	0.47 *	0.09	0.25
	Q	0.13	0.46 *	0.21	0.33	0.18

* = *p* < 0.05; ** = *p* < 0.01.

**Table 3:** Multicollinearity coefficients (ρ) for athlete self-report measures.

	Sleep	Fatigue	Soreness	Stress	Mood
Sleep	-	0.41	0.18	0.26	0.33
Fatigue	0.41	-	0.45	0.21	0.26
Soreness	0.18	0.45	-	0.11	0.11
Stress	0.26	0.21	0.11	-	0.52
Mood	0.33	0.26	0.11	0.52	-
